# Remote and semi-automated methods to conduct a decentralized randomized clinical trial

**DOI:** 10.1017/cts.2023.574

**Published:** 2023-06-07

**Authors:** Teresa Cafaro, Patrick J. LaRiccia, Brigid Bandomer, Helen Goldstein, Tracy L. Brobyn, Krystal Hunter, Satyajeet Roy, Kevin Q. Ng, Ludmil V. Mitrev, Alan Tsai, Denise Thwing, Mary Ann Maag, Myung K. Chung, Noud van Helmond

**Affiliations:** 1 Department of Anesthesiology, Cooper University Health Care, Camden, NJ, USA; 2 Cooper Research Institute, Cooper University Health Care, Camden, NJ, USA; 3 Won Sook Chung Foundation, Moorestown, NJ, USA; 4 Center for Clinical Epidemiology and Biostatistics Perelman School of Medicine University of Pennsylvania, Philadelphia, PA, USA; 5 The Chung Institute of Integrative Medicine, Moorestown, NJ, USA; 6 Cooper Medical School of Rowan University, Camden, NJ, USA; 7 Rowan University School of Osteopathic Medicine, Stratford, NJ, USA; 8 Division of General Internal Medicine, Cooper University Health Care, Camden, NJ, USA; 9 Division of Infectious Disease, Cooper University Health Care, Camden, NJ, USA; 10 Department of Family Medicine, Cooper University Health Care, Camden, NJ, USA

**Keywords:** Randomized clinical trial, remote methods, internet methods, electronic data capture, RCT, REDCap, decentralized

## Abstract

**Introduction:**

Designing and conducting clinical trials is challenging for some institutions and researchers due to associated time and personnel requirements. We conducted recruitment, screening, informed consent, study product distribution, and data collection remotely. Our objective is to describe how to conduct a randomized clinical trial using remote and automated methods.

**Methods:**

A randomized clinical trial in healthcare workers is used as a model. A random group of workers were invited to participate in the study through email. Following an automated process, interested individuals scheduled consent/screening interviews. Enrollees received study product by mail and surveys via email. Adherence to study product and safety were monitored with survey data review and via real-time safety alerts to study staff.

**Results:**

A staff of 10 remotely screened 406 subjects and enrolled 299 over a 3-month period. Adherence to study product was 87%, and survey data completeness was 98.5% over 9 months. Participants and study staff scored the System Usability Scale 93.8% and 90%, respectively. The automated and remote methods allowed the study maintenance period to be managed by a small study team of two members, while safety monitoring was conducted by three to four team members. Conception of the trial to study completion was 21 months.

**Conclusions:**

The remote and automated methods produced efficient subject recruitment with excellent study product adherence and data completeness. These methods can improve efficiency without sacrificing safety or quality. We share our XML file for researchers to use as a template for learning purposes or designing their own clinical trials.

## Introduction

Designing and conducting randomized controlled trials, the gold standard of research [1], appears to be out of reach for many institutions and independent researchers due to significant startup time, personnel requirements, study duration, and costs [2,3]. Enrollment in prospective clinical trials was significantly hampered due to pandemic restrictions on nonurgent contact with patients and reallocation of resources to COVID-19 (coronavirus disease 2019) care [4]. Technological advances in patient care via telehealth visits emerged rapidly and were promptly adopted by healthcare systems and reimbursement organizations [5,6]. As with clinical care, there was an urgent need to leverage technological advances to conduct prospective clinical trials remotely [4,7]. Decentralized trials, while not a new concept, were conducted in response to the pandemic [8–10]. Research Electronic Data Capture (REDCap) was also utilized by several groups to conduct their trials both prior to [11–14] and during the pandemic [15–17]. Our trial combined REDCap and other electronic applications to reduce many of the logistical requirements involved in executing a clinical trial.

During the coronavirus pandemic, we conducted a prospective randomized trial in healthcare workers on the protective effect of vitamin D supplementation on influenza-like illness and COVID-19 infection. The main trial design and main trial results can be found in van Helmond *et al*. [18]. We herein focus on the decentralized aspects of the study and describe electronic communication, electronic informed consent, study product shipping, and data capture methods to conduct a randomized controlled trial remotely using readily available software packages and platforms. We propose that these pragmatic methods can be used to efficiently conduct clinical trials without sacrificing safety or data quality. This article provides considerable detail and shares tools and an XML file containing our REDCap project metadata to be used as a guide, so one can save time in the development and conduct of their own decentralized study.

## Methods

### Study Overview

**Figure 1.**
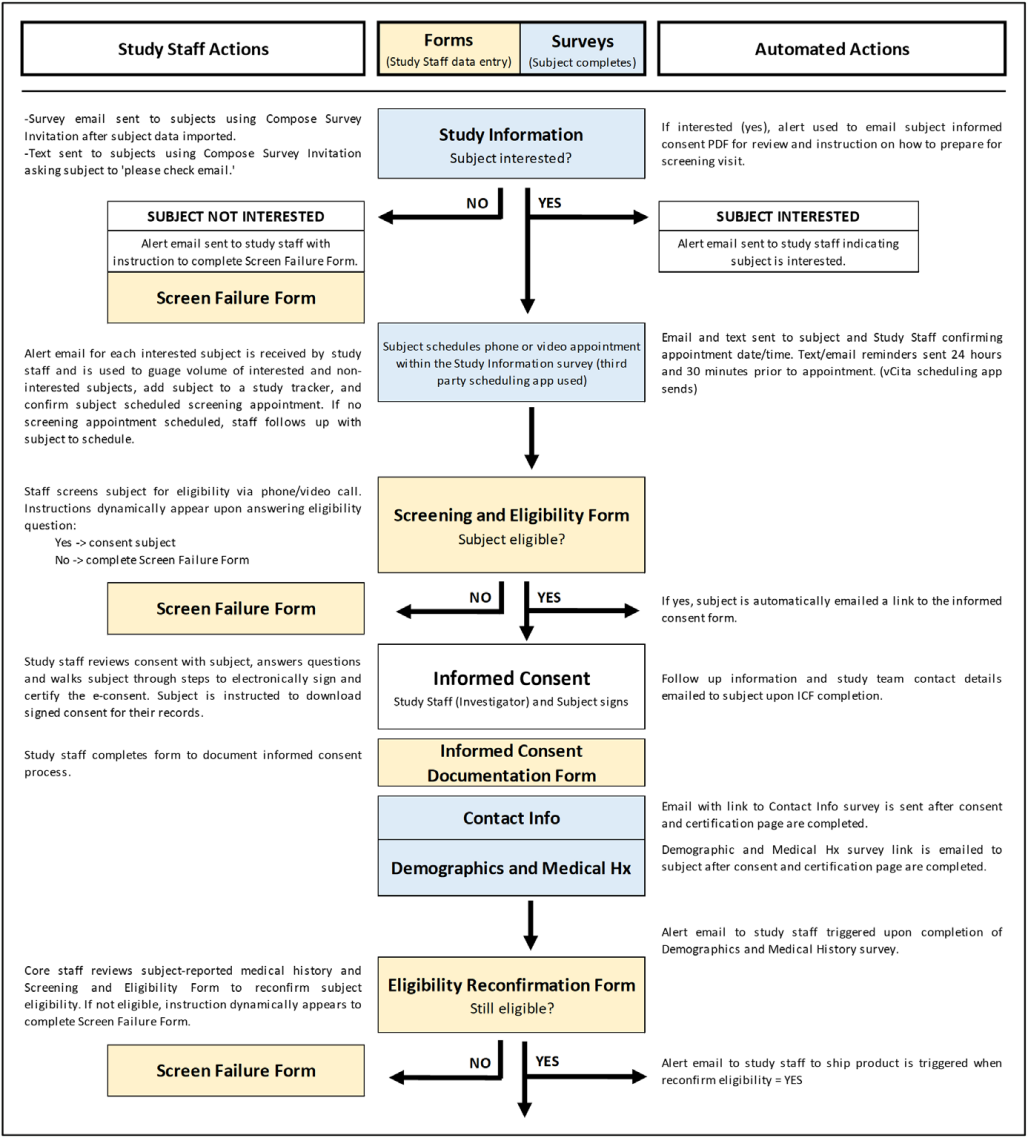
Surveys and forms flowchart – screening and enrollment period. REDCap instruments are used for data collection and can be created as forms to be completed by study staff (yellow) or surveys to be completed by subjects (blue). With institutional review board (IRB) approval and human resources (HR) support, healthcare employees were sent an invitation to participate. Employee names and emails provided by human resources were uploaded to the PHI form (protected health information) in order to send study invitations. The Study Information survey was created to invite employees to participate, allow potential subjects to schedule a phone or video screening appointment if interested, provide a summary of study details with text and video, and offer the full informed consent should potential subjects wish to review it. See Supplementary Figure 1 for more detail on the Study Information survey. Before sending the Study Information survey, emails were sent to all employees informing them of the upcoming study.

**Figure 2.**
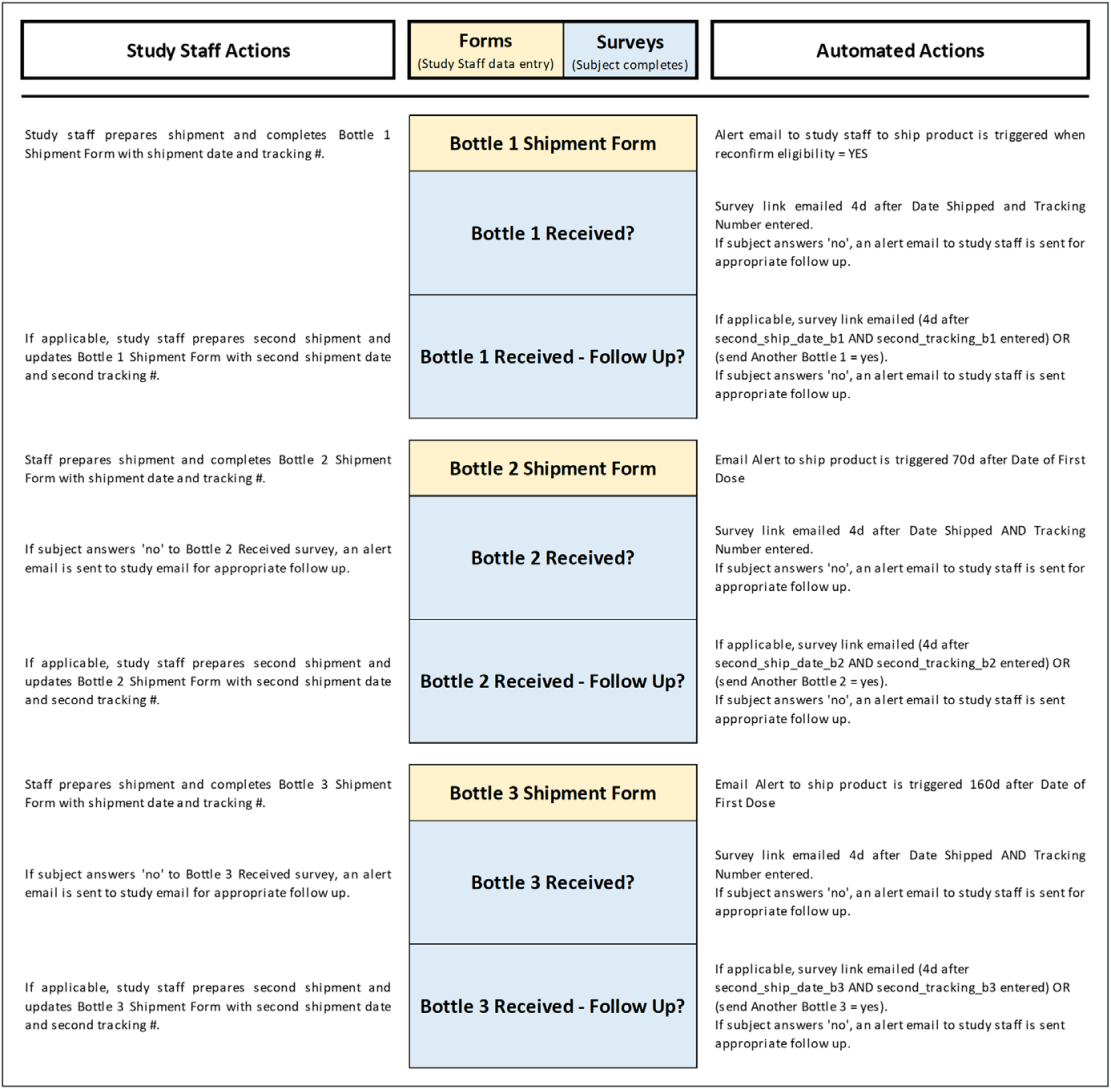
Surveys and forms flowchart – study product shipment and tracking. Study product was supplied to subjects in 3 separate bottles containing 90 gelcaps each (Bottle 1, Bottle 2, and Bottle 3) with the aim of easing pill counts for the subjects at month 3, month 6, and month 9 time points. The Bottle Shipment forms were created to capture shipment dates and carrier tracking numbers, to remind the staff member to include all items in the initial shipment (study product, pill organizer, and study packet letter), and to include special delivery instructions for the carrier, if any. Each Bottle Shipment form (see Panel d in Fig. 5 and Fig. 6) has piping in place (blue text) which displays subject shipping details on the form, thereby improving efficiency by removing the need to navigate to another form for the subject address. After an email alert instructing staff to ship product is received, study staff completes the Bottle Shipment Form and ships product. Four days after the shipment date, the “Bottle Received?” survey link is automatically sent to the subject. If subject indicates the bottle was not received (a “no” response), an email alert is sent to study staff prompting follow-up and completion of the second section of the Bottle Shipment Form with the replacement bottle shipment details, which will set off the same automated sequence described above for the replacement bottle being shipped.

**Figure 3.**
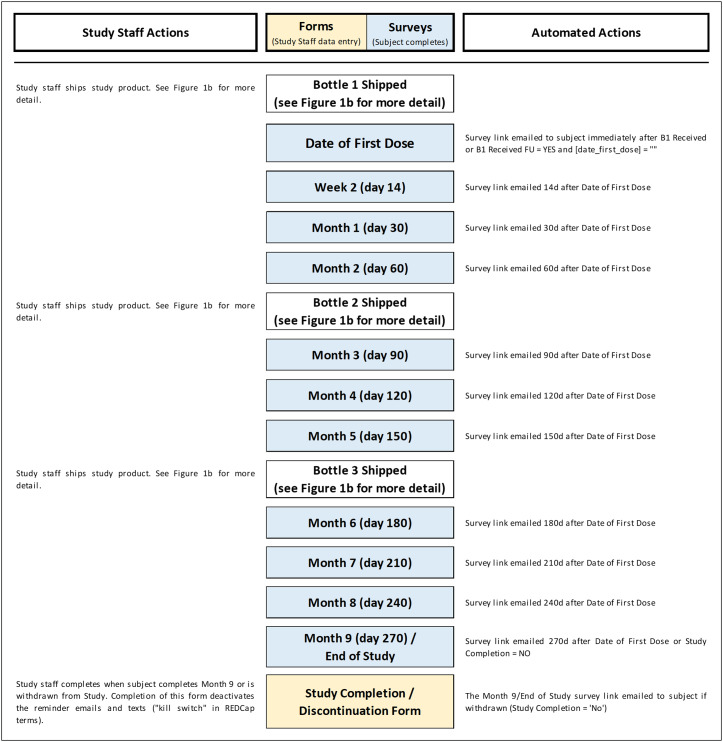
Surveys and forms flowchart – active study period. This flowchart illustrates the monthly surveys and the schedule by which the survey links are sent to subjects. REDCap automated survey invitations (ASI) are created for each survey and use conditional logic to send surveys at the specified time frame after the date of first dose. In addition, a “kill switch” was incorporated in the conditional logic for each ASI: if the Study Completion/Discontinuation Form is completed, the survey will not be sent. Example of conditional logic for Month 3 survey: datediff ([enrollment_arm_1] [date_first_dose], “today”,“d”, true) = 90 and [month_9_arm_1] [complete_study_eos] <> “0”. For more complex logic used for study product shipment and tracking, see Fig. 2. In addition, the XML file containing all conditional logic is shared in the Data Availability section.

Our trial was conducted in a large tertiary academic hospital and was institutional review board (IRB) approved prior to the enrollment of any subjects. All participants who received the study supplement (vitamin D3) provided electronic informed consent. All study information and trial logistics were managed using the Research Electronic Data Capture (REDCap) system version 10.8.2 developed by Vanderbilt University [19,20]. The REDCap automated survey invitations (ASIs), branching logic, piping feature, and alerts were heavily utilized for automation along with integrated applications for texting and appointment scheduling. All study contacts with subjects, including recruitment, consent, data collection, and safety monitoring were virtual, with the exception of clinically indicated outpatient visits. An overview of all remote procedures and semi-automation is provided for recruitment, screening, and enrollment in Fig. 1, for study product shipping and tracking in Fig. 2 and for the general study flow of surveys and forms in Fig. 3. Additionally, the XML file for this trial and other tools are shared as supplements.

### Randomization

All hospital employees (subjects) were randomly assigned study numbers and divided into two groups: 1) the intervention group (vitamin D supplementation) and 2) the control group. Randomization was completed using the randomization function in Microsoft Excel. The intervention group was approached for study participation. The control group did not receive placebo and was approached for voluntary survey completion toward the end of the 9-month intervention study period to compare the two groups for demographic and clinical characteristics. Main trial details can be found in van Helmond *et al.* [18].

### Recruitment

#### Intervention and control groups

As recruitment by email can be challenging, we opted to inform subjects of the study prior to sending the REDCap-generated email containing the study invitation link. First, a bulletin summarizing the study and informing employees of potential contact was posted on our institutional intranet. Subjects were made aware of the posting via the hospital’s weekly email announcement from institutional leadership. Second, the principal investigator emailed subjects informing them the forthcoming study invitation email was not fraudulent and the study invitation link was safe to open. The study was led by healthcare system staff; and participation was voluntary.

As employees are a known vulnerable study population, extra measures were taken to ensure the subjects did not feel coerced in any way. Each of the three aforementioned notices (bulletin post and two emails) to the employees included “participation is completely voluntary” statements. In addition, the first invitation survey (the Study Information survey) stated their employment and/or standing with the institution would not be affected in any way if they chose to participate or if they chose to decline. Further, the informed consent, which was reviewed in detail with each subject, contains statements detailing (1) participation is voluntary; (2) they may quit at any time; and (3) their decision to participate or not participate would not affect their employment nor their care at the institution.

### Screening and Electronic Informed Consent

#### Intervention group

The intervention group subjects were emailed a link to the Study Information survey through REDCap. The survey links were sent to each employees’ work email via our secure institutional email server. This survey contained summarized study information, a brief informational video about the study, and a full PDF of the informed consent. Subjects were asked to indicate their potential interest in the study (Supplementary Figure 1). If interested, instructions dynamically appeared for the subjects to electronically schedule their screening and consent appointment (Supplementary Figure 2). We used a third-party Health Insurance Portability and Accountability Act (HIPAA)-compliant, automated appointment scheduling system (vCita, Bellevue, WA, USA) developed for telemedicine appointments. The scheduling application was integrated within REDCap using an application programming interface (API). Both telephone and video call (Zoom Video Communications, San Jose, CA, USA) appointments were offered. The scheduling application allowed our study team members to adjust their availability for appointment assignments. After a potential subject booked a screening appointment, both the subject and study team member were sent an automated calendar invitation for the appointment with the relevant contact information or video call link. In addition, the study team member was informed of the scheduled appointment via text in real time. Through the scheduling application, the potential subject was automatically sent a PDF copy of the informed consent form (without signature section) for their review before the screening appointment as well as instructions on how to prepare for the screening appointment. Instructions for the appointment included reviewing the informed consent form, having information on any medications or supplements they were taking available, and being seated at a computer/tablet/smartphone with access to internet and email. The scheduling application was set to send reminder emails 24 hours and 30 minutes prior to each appointment.

**Figure 4.**
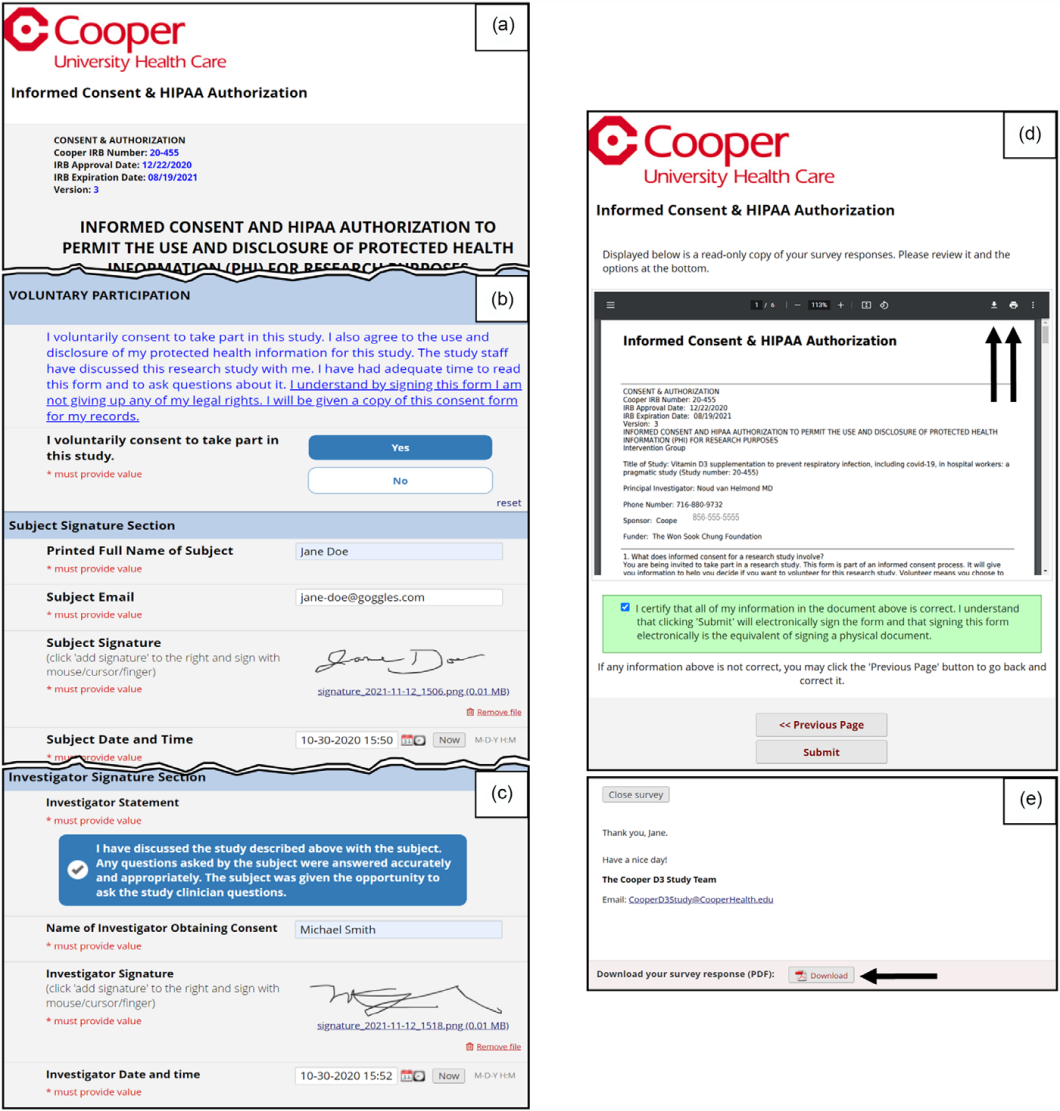
Electronic informed consent (e-consent). Panel a: the e-consent process was approved by our local IRB. The REDCap electronic consent framework (an option in survey settings) was utilized to consent and collect signatures from subjects and study staff remotely. The survey formatting was modified from the REDCap shared library. Panel b: the subject reads the voluntary consent statement, indicates if they voluntarily consent to participate, provides signature, email, and date/time, and saves the document to allow for the investigator to sign and date/time the consent. Panel c: the investigator reviews and checks the investigator statement, enters name, provides signature and date/time, and saves entries. The subject refreshes the webpage, scrolls to bottom, confirms both subject and investigator signatures, and clicks “Next page” (not shown). Panel d: the consent framework includes a separate certification page. The subject is asked to review the inline signed consent, instructed on how to download or print the signed consent, and asked to certify that details are correct. Upon submitting the survey, a PDF of the consent is automatically archived in the file repository (not shown). Panel e: after submission, subject had a second opportunity to download PDF of signed consent. Immediately following informed consent, subjects were emailed study team contact information, a link to the Demographics and Medical History form and a link to the subject Contact Information form. Supplement 1 is available detailing the steps the subject and investigator took to electronically sign the informed consent.

During the scheduled appointment, a study team member described the study in detail. If a subject confirmed interest, eligibility was determined through a screening and eligibility script which focused on exclusion criteria related to medical history and medication use. During the call, the study team member completed a screening and eligibility form in REDCap (Supplementary Figure 3). Depending on the information entered, relevant instructions dynamically appeared for the team member (e.g., if eligibility = no, subject is a screen failure; proceed to the Screen Failure form to record pertinent information). Eligible subjects who wished to proceed with the consent interview (e.g., eligibility = yes) were automatically sent an email with a link to the subject-specific informed consent. Subjects were asked to open the informed consent form from their email on a computer, tablet, or smartphone. The informed consent form was reviewed in detail, and teach-back questions were used to confirm the subject’s understanding of the study. After reviewing the consent and answering any questions, the study investigator instructed the subject on the steps needed to electronically sign and date-timestamp the informed consent form. Supplement 1 describes the detailed steps of the electronic consent signing process. The electronic consent was adapted from Combined Consent & HIPAA Authorization Template as posted in the REDCap library [21]. We used the electronic consenting framework in REDCap (Fig. 4) which has an Auto-Archiver and e-Consent Framework wherein a PDF of the signed consent is automatically archived in REDCap’s file repository. The subjects’ name and consent version were automatically included on the footer of each page as extra documentation of the identity of the subject who is consenting. Subjects had two opportunities to download the signed informed consent form during the process, which are also described in Supplement 1. Alternatively, subjects were emailed a fully signed copy of the informed consent form through secure email.

#### Control group

Immediately after signing the consent, subjects proceeded to answer survey questions regarding demographics, medical history, use of vitamin D supplementation, vaccination history, and concomitant medication.

### Study Product Shipping

#### Intervention group only

**Figure 5.**
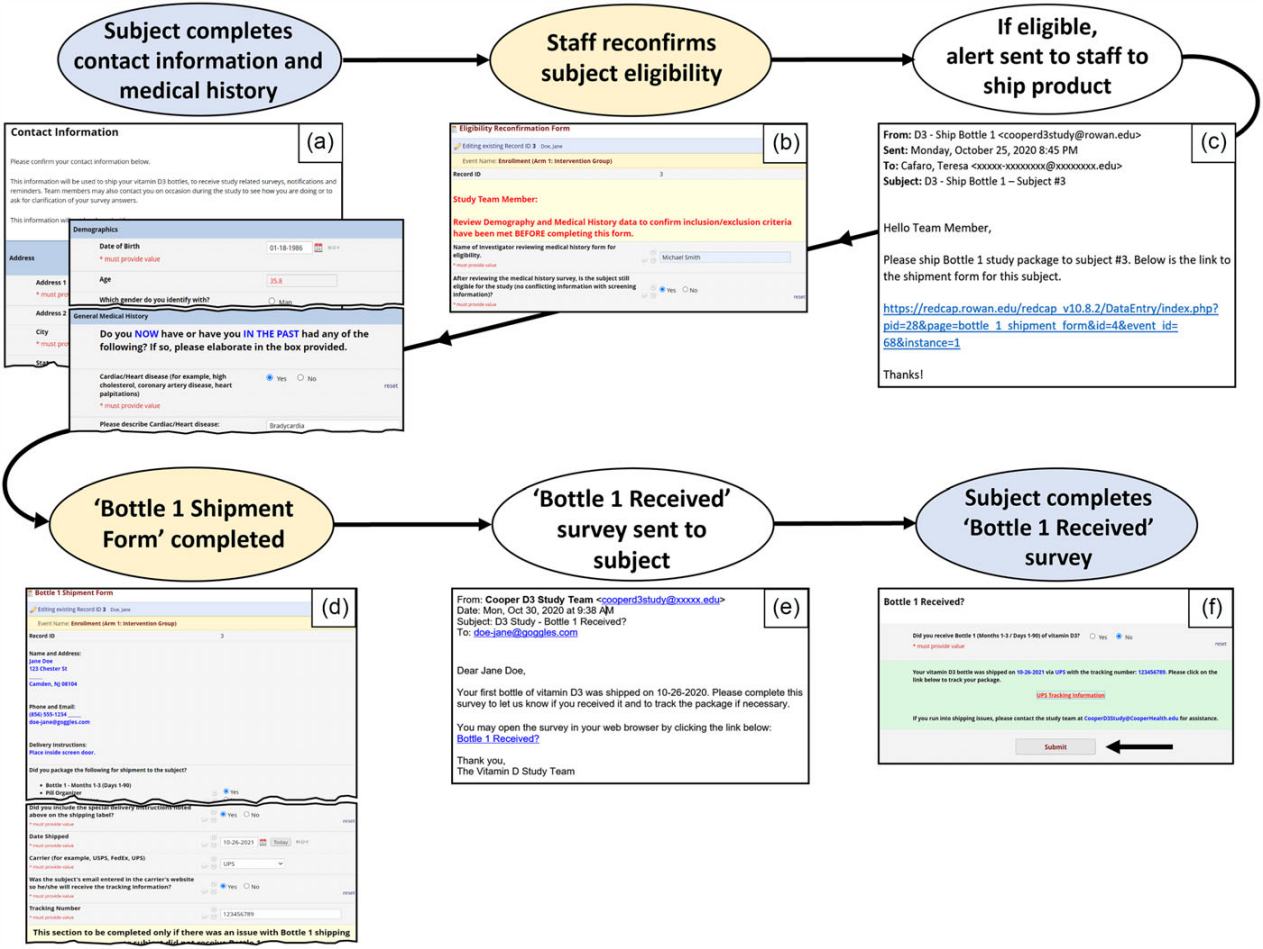
Automated enrollment steps. (Blue = survey; yellow = form; white = automated action). This schematic details the steps after consent is signed. After consent is signed and submitted, subject is auto-sent two emails with survey links using Automated Survey Invitations (ASI): Contact Information survey and Demographics and Medical History survey (Panel a). Once subject completes the surveys, an alert email (not shown) is triggered to core study staff with instruction to review medical history and reconfirm subject eligibility (Panel b). If not eligible (= no), dynamic instructions appear to complete the Screen Failure form (not shown). If eligible (= yes), an alert email with a link to the Bottle 1 Shipment form is automatically sent to study staff notifying them to ship the first bottle of study product (Panel c) and complete the Bottle 1 Shipment form (Panel d). The Bottle 1 Shipment form has piping in place (blue text) which allows the staff member to see the subject shipping details on the form, thereby improving efficiency by eliminating the need to navigate to another form. Four days after product is shipped, an automated survey invitation (Panel e) is sent to the subject to request completion of the Bottle 1 Received survey (Panel f). If the package was not received (= no), the survey dynamically provides the shipment tracking link for the subject and subsequently an alert email is sent to study staff informing them of shipping issue (continued in Fig. 6).

**Figure 6.**
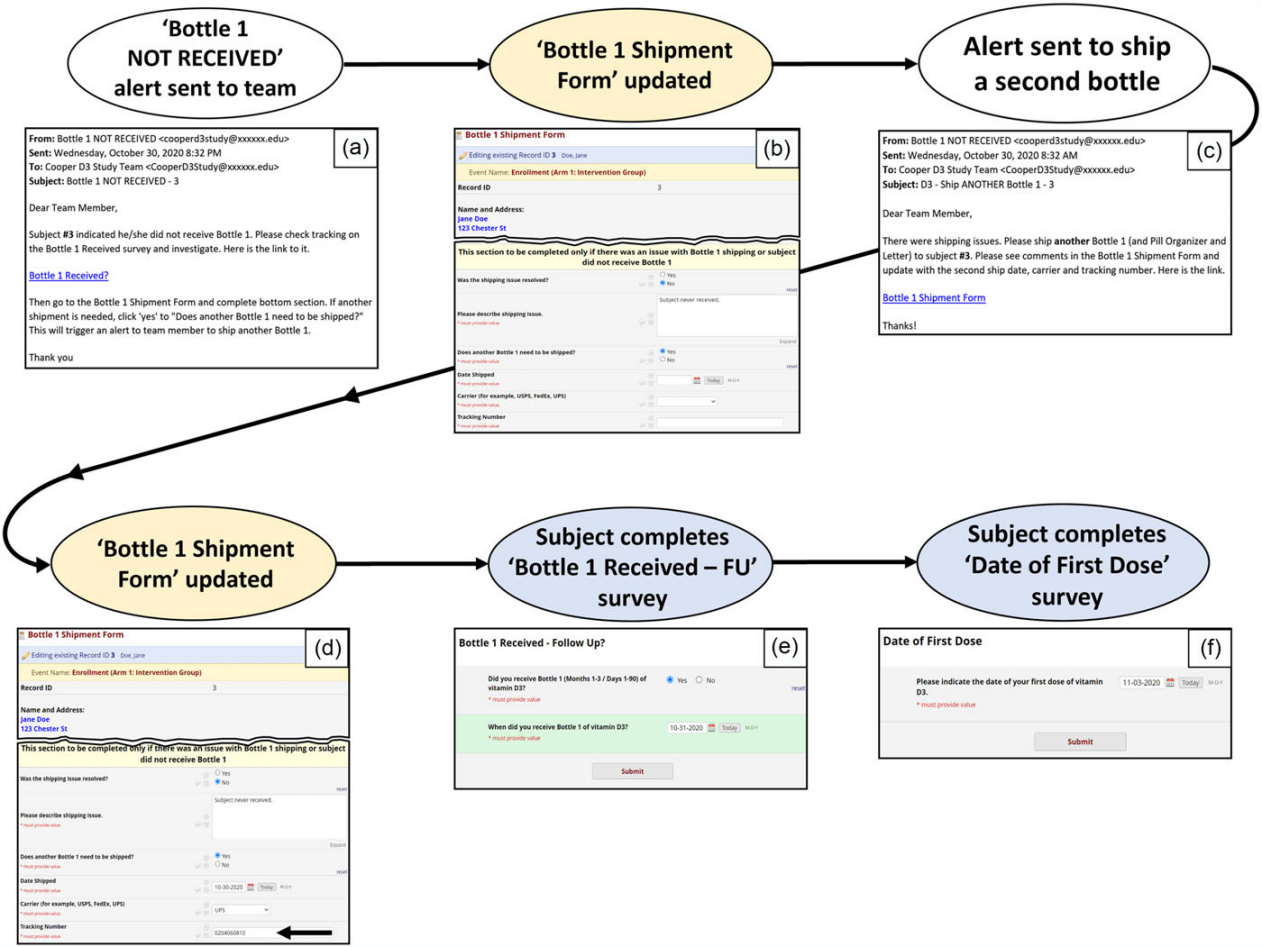
Automated enrollment steps. (Blue = survey; yellow = form; white = automated action) (cont.). If bottle was not received, an alert email was sent to study staff informing them of a shipping issue and directing them to investigate (Panel a). After staff confirmed a second bottle must be shipped, they answered the first three questions of the last section of the Bottle Shipment form (Panel b). By answering “yes” to “Does another Bottle 1 need to be shipped?” an alert (Panel c) was automatically sent to designated study staff requesting they ship another bottle and complete the date shipped, carrier, and tracking number fields on the Bottle 1 Shipment Form (Panel d). Four days after product was shipped, an automated survey invitation was sent to the subject requesting completion of the Bottle 1 Received – Follow-Up survey (Panel e). If the package was received (= yes), a question asking for the date received dynamically appeared. Once submitted, the Date of First Dose survey was immediately triggered and sent to the subject. A study staff member monitored completion of Date of First Dose surveys dose and followed up with subjects to request completion. Thereafter, surveys were automatically sent as scheduled using the date of first dose as shown in Fig. 3.

The automated study product shipment details are depicted in Fig. 2, Fig. 5, and Fig. 6. Study product was provided to subjects in three separate shipments over the 9-month study period. The first shipment was a study packet including 1) a 90-day supply of study supplement; 2) a study packet letter providing study information and instructions; and 3) a 7-day pill organizer to aid in adherence to study product. The study packet was shipped upon enrollment, and two subsequent bottles were shipped at 3-month intervals. Shipment alerts were automatically sent to study staff based on the subjects’ study day threshold (i.e., day 0, day 90, and day 180) and if their study status remained active.

When shipment notifications were received by study staff, subjects’ information was entered in the carrier’s electronic shipping form which created tracking numbers and address labels. Tracking numbers were entered into the REDCap shipping form along with date shipped and the person shipping the product. Labels were applied to premade packages that were then delivered to the shipping company. All notifications and alerts for shipments or repeat shipments were through REDCap.

Four days after product shipped, an automated “Bottle Received?” survey was emailed to subjects. A link to the shipment tracking number dynamically appeared to subjects if they answered “no” on the survey. Answering “yes” triggered the Date of First Dose survey link to be sent to subjects. Because the shipment tracking link was only visible on the survey if subjects answered “no,” the shipment tracking link was later inserted into the Bottle 2 and 3 shipment *forms* that staff completed and often referenced. The sample shipment tracking link utilized HTML coding:

<a target=“_blank” href="https://www.ups.com/track?loc=null&tracknum=[month_2_arm_1][tracking_b2] &requester=WT/">UPS Tracking Information</a>

### Survey Data Collection

#### Intervention group

At 2 weeks, 1 month, 2 months, 3 months, 4 months, 5 months, 6 months, 7 months, 8 months, and 9 months after date of first dose, an adherence and safety survey was sent as a link via email. ASIs were utilized to email subjects links to each survey at the study-defined time points (14 days, 30 days, 60 days, 90 days, etc).

Adherence to survey completion is necessary for robust data collection; thus, we employed methods to increase this adherence. If the survey was not completed within 24 hours, an automated reminder was sent daily for up to 3 days. If an individual survey was not completed after three reminders, a study team member followed up with the subjects via email, text, and/or phone. If follow-up emails or texts were sent, the link to the survey was included for the subjects’ convenience and to increase adherence.

The Month 9/End of Study survey was sent to subjects upon completion of the 9-month study period (270 days) or upon study withdrawal. In addition, staff completed the Study Completion/Discontinuation Form to update each subject’s study status as completed or withdrawn. The answer to the question, “Did the subject complete the study?” was critical, as it served several important functions. A “no” response: (1) triggered the Month 9/End of Study survey link to be emailed to withdrawn subjects; (2) prevented remaining monthly surveys from being sent to the withdrawn subject; (3) stopped future study product shipment alerts from being sent to study staff; and (4) prevented study product reminders from being sent to subjects (discussed below). A “yes” response discontinued study product reminders to subjects.

Alerts and ASIs with conditional logic were utilized throughout the REDCap project to control when or if a survey or alert was to be sent. This conditional logic used for the purposes above is considered “stop logic” or a “kill switch” in REDCap. An example of the Month 6 ASI conditional logic below tells REDCap to send the Month 6 survey if it has been 180 days since first dose *and* if the Study Completion/Discontinuation Form question, “Did the subject complete the study?” is yes or blank. For access to all forms, surveys, and conditional logic, see Data Availability section.

datediff([enrollment_arm_1][date_first_dose], ‘today’,’d’, true) = 180 and [month_9_arm_1][complete_study_eos] <> ‘0’

#### Control group

Toward the end of the 9-month intervention study period, survey invitations were sent using the Compose Survey Invitations feature in REDCap via the Survey Distribution Tools. No further information was required from control subjects; however, some subjects were contacted to obtain clarification on questionable data.

### Study Product Adherence

#### Intervention group only

Several methods were implemented to increase study product adherence, including reminder emails and texts sent twice weekly, estimated self-reported vitamin D adherence on every survey, self-reported pill counts, and adherence reports for study staff review. Circular pill boxes (Pill Thing, Ellisville, MO. USA) were provided with compartments for each day of the week [22] (Supplementary Figure 4). Reminder emails included a photo of vitamin D gelcaps for picture association, and automated reminder texts were sent using a text messaging service (Twilio, San Francisco, California, USA) that was integrated with REDCap. Subjects were asked to estimate the number of pills they missed on the monthly surveys, and at 3-month intervals subjects were asked to count and report the pills remaining in the study product bottles. Adherence reports in REDCap showing the self-reported estimations and pill counts were monitored; any subjects with less than 70% adherence rates were contacted to determine errors or other reasons for low adherence rates.

REDCap’s ASIs and surveys were used unconventionally to send email and text reminders twice weekly throughout the 9-month period. Multiple surveys were created, but only the ASI emails and texts were used to send the message, “This is a friendly reminder to take your vitamin D_3_ capsule each and every day.” The message did not include the survey link, a capability offered by REDCap ASI. Each ASI allows one invitation and a maximum of five reminders to be sent at increments of one’s choosing. Seven-day increments were chosen; thus, one reminder could be sent weekly spanning 42 days. In order to send subjects two reminders (one text and one email) twice weekly, a total of 28 surveys were required to span the 270-day study period. An example of the ASI conditional logic for one of the many reminders is below.

[enrollment_arm_1][date_first_dose] <> “” and [month_9_arm_1][complete_study_eos] = “” and datediff([enrollment_arm_1][date_first_dose], ‘today’,’d’, true) > 125 and datediff([enrollment_arm_1][date_first_dose], ‘today’,’d’, true) < 168

### Safety and Adverse Event Monitoring

#### Intervention group only

**Figure 7.**
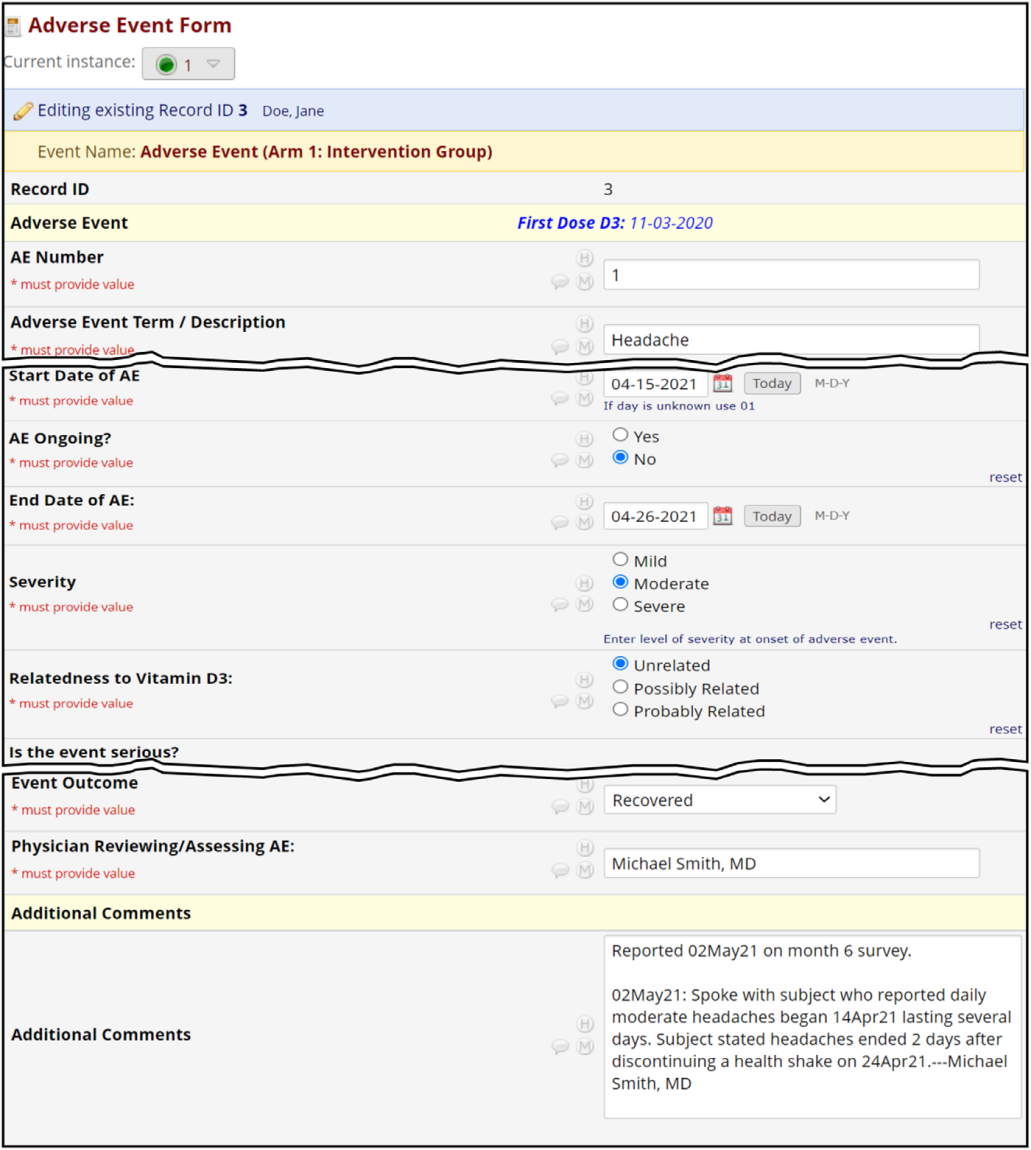
**Safety monitoring/adverse events.** This is a typical adverse event (AE) form that collects event term, start/end dates, severity, relatedness, seriousness, and event outcome. Direct data entry was employed in lieu of paper source documentation. The “Additional comments” field was used to document communications with the subjects. The “Physician reviewing / assessing AE” field was included to document physician review, relatedness assessment, and adjudication. Prior to physician adjudication, this field was utilized for study team tracking to indicate which study team member was following up on a particular AE; once the AE was ready for physician review and relatedness assessment, “Complete” was entered to signal the physician to review, assess relatedness of, and adjudicate the AE. A weekly AE report derived from REDCap was shared with team members to track progress in AE monitoring.

To monitor subject safety, monthly surveys were designed to screen for potential vitamin D toxicity symptoms such as hypercalcemia and nephrolithiasis. Vitamin D laboratory test levels were not included as the safety profile for vitamin D_3_ at a 5,000 IU dose has been shown to be safe [23–27]. The surveys also included questions regarding new or unusual health changes, new medications, and pregnancy. If any of the survey safety questions were answered “yes,” email alerts with links to the survey were triggered to the study team which enabled prompt follow-up with subjects and adverse event (AE) reporting. A hospitalization question was also included in the surveys which, if answered “yes,” triggered a separate serious adverse event (SAE) alert email to staff. If indicated, a clinic visit was offered, or laboratory tests were ordered to rule out hypercalcemia or nephrolithiasis. On each AE form (Fig. 7), documentation of physician review and assessment of the AEs was direct data entered into the “Physician Reviewing/Assessing AE” field. Documentation of subject communications were also direct data entered into the “Additional Comments” field in each AE form; this field served as a charting tool. In addition, AE reports were produced weekly using REDCap’s reporting module and were distributed by email to team members to track AEs for follow-up.

A Subject Summary form (Supplementary Figure 5) was created which pulled (or piped) pertinent subject data from other forms/surveys to provide study staff with essential information such as phone number, date of first dose, study status, medical history, survey data, and AEs with all additional comments. Staff utilized this time-saving summary prior to and during follow-up phone calls with subjects. The subject summary tool was also used for quick miscellaneous reference as needed.

### System Usability

The System Usability Scale (SUS) is a well-recognized and standardized tool to assess perceived usability of systems [28]. The SUS survey was sent as a REDCap survey well into the maintenance period of the study and after 57 intervention subjects completed the study. The survey was sent to 260 enrolled intervention subjects (299 less 39 subjects who had withdrawn consent). In addition, a separate SUS survey was sent to 9 of the 10 study team members to score usability from their perspectives with a focus on system usability during the recruitment, screening, and informed consent process. The SUS subject survey is available in the shared XML file – see Data Availability section.

## Results

The time from conception of the trial (March 2020) to study completion (November 2021) was 1 year, 9 months (21 months).

### Screening, Electronic Consent, and Enrollment

Two hundred and ninety-nine intervention subjects were enrolled in the study over a 3-month period. Ten study staff members screened and consented subjects; they contributed a combined estimated total of 225 hours for the screening and consenting process. Electronic consenting procedures were seamless and did not require paper printing of any consent form. Five hundred and seventy-eight control subjects voluntarily completed the control survey. Data regarding the distribution of hospital workers’ job titles were collected and are displayed in Table 1.

**Table 1.** Participant job title distribution

	Intervention (*n* = 231)	Control (*n* = 578)
Job title, *n* (%)		
Doctor	24 (10)	100 (17)
Nurse practitioner/physician’s assistant	14 (6)	42 (7)
Nurse	57 (25)	130 (22)
Medical technologist	9 (4)	18 (3)
Respiratory therapist	4 (2)	2 (0)
Housekeeper, inpatient nutritionist, medical social worker, pastor/chaplain, psychologist	8 (3)	13 (2)
MRI/ultrasound/radiology technologist	5 (2)	15 (3)
Physical therapist	2 (1)	4 (1)
Occupational therapist	0 (0)	1 (0)
Administrative professional	44 (19)	94 (16)
Other	64 (28)	159 (28)

### Study Product Shipping

Only two study team members were responsible for study product shipment. Very few emails outside of REDCap alert notifications were needed when expected shipping issues arose. Out of 829 shipments, only 17 required a second bottle to be shipped. Most reasons for second shipments were carrier delays or lost shipment; other reasons included address changes and damaged product.

### Data Completeness

The average adherence rate for completing monthly surveys was 98.5%; the high rate is attributed to automated reminders and manual follow-up via REDCap report generation and review for missed surveys. A dedicated staff member manually followed up with emails, texts, and/or phone calls if subjects did not complete surveys after the initial ASI and three daily reminder emails were sent. Throughout the 9-month study period, a total of 458 texts and 616 emails were sent, and 208 phone calls were made to boost survey adherence.

### Study Product Adherence

The average self-reported vitamin D adherence rate was 87%. We believe this was due to the provision of a pill organizer, our twice weekly email and text reminders, our monthly survey question regarding number of missed doses, and our every third-month survey question regarding number of pills left in the bottle (Supplementary Figure 4).

### Safety Monitoring

The automated alerts, direct data entry of subject communications, the subject summary tool, and weekly AE reports were the tools that improved efficiency and safety data accuracy and allowed 3 team members to effectively monitor and follow 388 AEs over the course of the study. Three in-person clinic visits occurred, and 22 laboratory tests were ordered as a result of AEs follow-up.

### System Usability

SUS survey data assessed system usability from both the subject and study staff perspectives. Out of 260 subject surveys sent, 196 subjects responded with an average score of 93.8%. All nine staff members who were sent the SUS survey responded with an average score of 90% when considering the usability during recruitment, screening, and electronic consent.

## Discussion

### Principal Findings

The internet methods developed to conduct a randomized interventional controlled trial allow for efficient subject recruitment, safety data collection, and other trial-related tasks with excellent study data completeness. Our trial was designed to be conducted remotely utilizing the REDCap data capture platform and vCita electronic scheduling application.

Remote and automated electronic methods were utilized by a group of 10 study staff to successfully and efficiently recruit, consent, and enroll 299 intervention subjects. Each of the 10 team members contributed varying hours to the screening and consenting process; none of the members was dedicated full-time to this process. The total estimated number of hours contributed by the research team during screening and enrollment was 225 hours. Job title data presented in Table 1 describe the distribution of hospital employee jobs. By applying complex conditional logic in REDCap, efficient automation of surveys, alerts, product shipment notifications, and study product reminders was achieved. In addition to receiving high system usability scores (SUS), the methods used produced excellent adherence rates in both subject study product compliance and survey completion.

Decentralized clinical trials of assorted designs were conducted during the pandemic and offered various strategies for safety data collection [8–10]. Josan *et al*. conducted a 6-month trial collecting AEs via monthly televisits [8]. Kaizer *et al.* used safety triggers sent to staff for telephone follow-up when daily surveys were not completed for 2 consecutive days on their short-duration trial [9]. Naggie *et al.* ran a short-duration trial and asked participants to complete assessments daily through a study portal [10]. Specifics of how their safety data was captured and other electronic methods were lacking.

Our approach to safety monitoring and the general automated trial flow offered fail-safe and efficient methods in data retrieval. Potential AEs or SAEs were presented via alerts triggered from affirmative survey responses allowing immediate follow-up with the participant. The email alerts contained links that saved staff time navigating to the survey in question. Other innovations improved efficiency, such as utilizing the “Physician Reviewing/Assessing AE” field for different purposes, improving the process of AE follow-up by staff, then ultimately signaling the physician to review and assess AE relatedness (Fig. 7). Paper source documentation on safety data was eliminated by using an “additional comments” field in the AE form to document discussions with subjects via direct data entry. This documentation provided up-to-date information to any staff accessing an AE. Finally, the Subject Summary form greatly aided staff in preparing for participant follow-up by presenting details such as name, phone number, medical history, survey responses, and previous AEs, eliminating the need for navigation to other forms and surveys (Supplementary Figure 5).

There are other noteworthy aspects of decentralized studies. Facility space to interview subjects was unnecessary as all screening and consenting were performed remotely. Storage space for all the typical paperwork was greatly reduced. Additional savings are noted in that staff, and subjects did not need to travel to and from a facility. Electronic consenting saved on staff that would have been needed to review and archive paper consents. Time was also saved with e-consenting because the auto date- and time-stamping feature in REDCap eliminated errors that are common with traditional paper informed consents [29].

The electronic tools and automation developed in REDCap for data collection, safety monitoring, and overall study maintenance reduced the time typically necessary to conduct certain trial-related tasks. We show improved efficiency by comparing our automated tasks to manual tasks associated with in-person trials in Supplement 4. The startup time and effort in our remote trial development were considerable and not without its challenges. Startup time is a strong factor in the efficient conduct of clinical trials [3]. We stress these efforts do not have to be reinvented. For example, to facilitate implementation of our consenting methods we have provided in Supplement 1 step-by-step instructions on how to seamlessly sign the electronic consent remotely. We suspect researchers who use our tools and our XML file along with other shared practical examples [15] would reduce their trial development time and time to conduct their trial.

## Limitations/Future Directions

All of our subjects are functionally English-speaking employees, and we did not have the need for translation services. REDCap does have a Multi-Language Management application that can be utilized for other studies that include non-English-speaking subjects. Video surveys are a possibility to address the participation of low-literacy individuals.

The IRB approved all surveys and consent form. In addition, we used teach-back questions during the consent process to ensure subjects understood the study procedures, risks, and benefits. In retrospect, we see there could be improvements made to reduce some of the medical terminology used in the medical history and the monthly surveys. In future studies, we will aim to improve consent form and survey language.

We encountered challenges with recruitment that resulted in low enrollment numbers. We surmised that many potential participants were lost due to the incompatibility between outdated browser software and the third-party scheduling application. Although our institutional IT department confirmed all browser software had been updated with the latest version, we found that individual subjects were still using the outdated browser on their desktops which caused problems with electronically scheduling screening appointments. Other lessons learned are described in Supplement 2.

Many users find creating a project in REDCap daunting. REDCap is being used in innovative ways for purposes other than research among institutions [30]. Many articles are available that share detailed information and data such as REDCap conditional logic [17], trial flowcharts and lessons learned [13,16], and practical examples such as XML data files [15] which will give a novice user a complete REDCap project to use as a template to develop their own projects with ease.

The efficiencies described above are the result of extensive preparation and coordination efforts in the development of the remote methods for our trial.

Access to the XML file for this project is described in the Data Availability section.

## Conclusions

The remote and automated methods developed for this randomized clinical trial yielded efficient subject recruitment with excellent study data completeness and intervention adherence without sacrificing safety or quality. These shared methods, tools, and XML file offer researchers the prospect of reducing the time to develop and conduct their own clinical trials.

## Data Availability

The datasets generated during and/or analyzed during the current study are available from the corresponding author following reasonable request. An XML file containing our REDCap project metadata is available for upload into the reader’s own REDCap application (Supplement 3). Subject data is not included in this XML file. The metadata includes user roles, record status dashboards, reports, alerts and notifications, surveys and survey settings, and ASIs. We share this file to help other researchers observe how REDCap was used in our trial, learn from it, or use it as a starting point in creating their own study. The XML file can be uploaded simply by following these steps: (1) download the XML file; (2) create a new project in REDCap; (3) in the “Project creation option” select “Upload a REDCap project XML file”; and (4) select “Choose File” and locate and open the XML file that was downloaded in Step 1.

## Supplementary material

For supplementary material accompanying this paper visit https://doi.org/10.1017/cts.2023.574. click here to view supplementary material
