# Supplementary material for: Remote and semi-automated methods to conduct a decentralized randomized clinical trial
Source: J Clin Transl Sci. 2023 Jun 7;7(1):e153. doi: 10.1017/cts.2023.574 (PMC10388435; doi:10.1017/cts.2023.574)
Supplement: Supplementary file 1 [file S2059866123005745sup001.zip › suppl_data/S2059866123005745sup001.pdf]

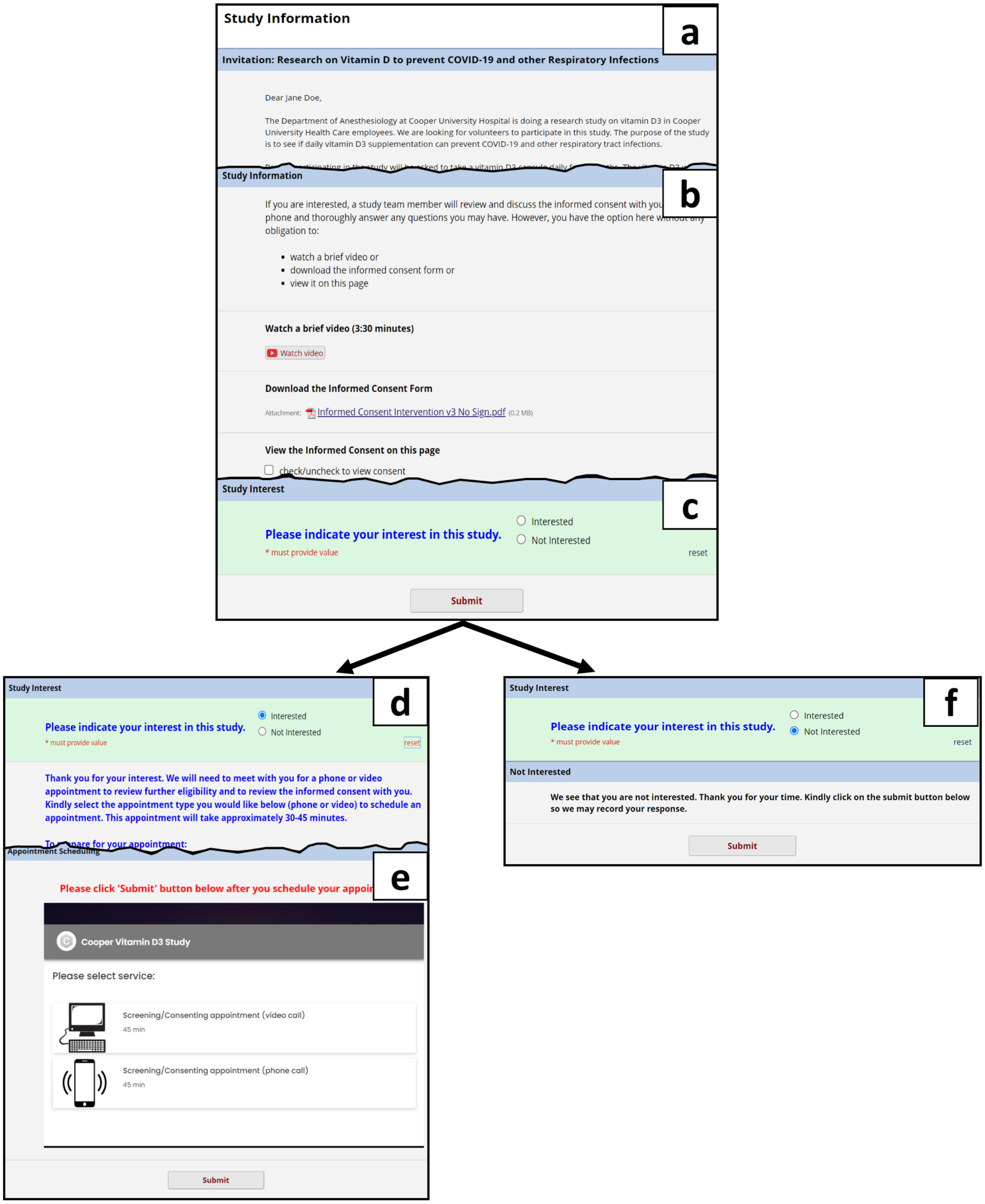

Supplementary Figure 1. Study information survey.

Panel a: Invitation with brief explanation. Panel b: Study information with option to watch brief video, download consent, or view consent inline. Panel c: Study interest question which, depending on the answer, will dynamically reveal next steps and relevant instructions. Panel d: If interested, instructions for screening appointment preparation and electronic appointment scheduling dynamically appear. Panel e: Electronic scheduler integrated with REDCap using API. See Supplementary Figure 2 for more detail on electronic scheduler. Panel f: If not interested, a thank you to the subject with a request to submit survey dynamically appears.
