# Supplementary material for: Remote and semi-automated methods to conduct a decentralized randomized clinical trial
Source: J Clin Transl Sci. 2023 Jun 7;7(1):e153. doi: 10.1017/cts.2023.574 (PMC10388435; doi:10.1017/cts.2023.574)
Supplement: Supplementary file 1 [file S2059866123005745sup001.zip › suppl_data/S2059866123005745sup002.pdf]

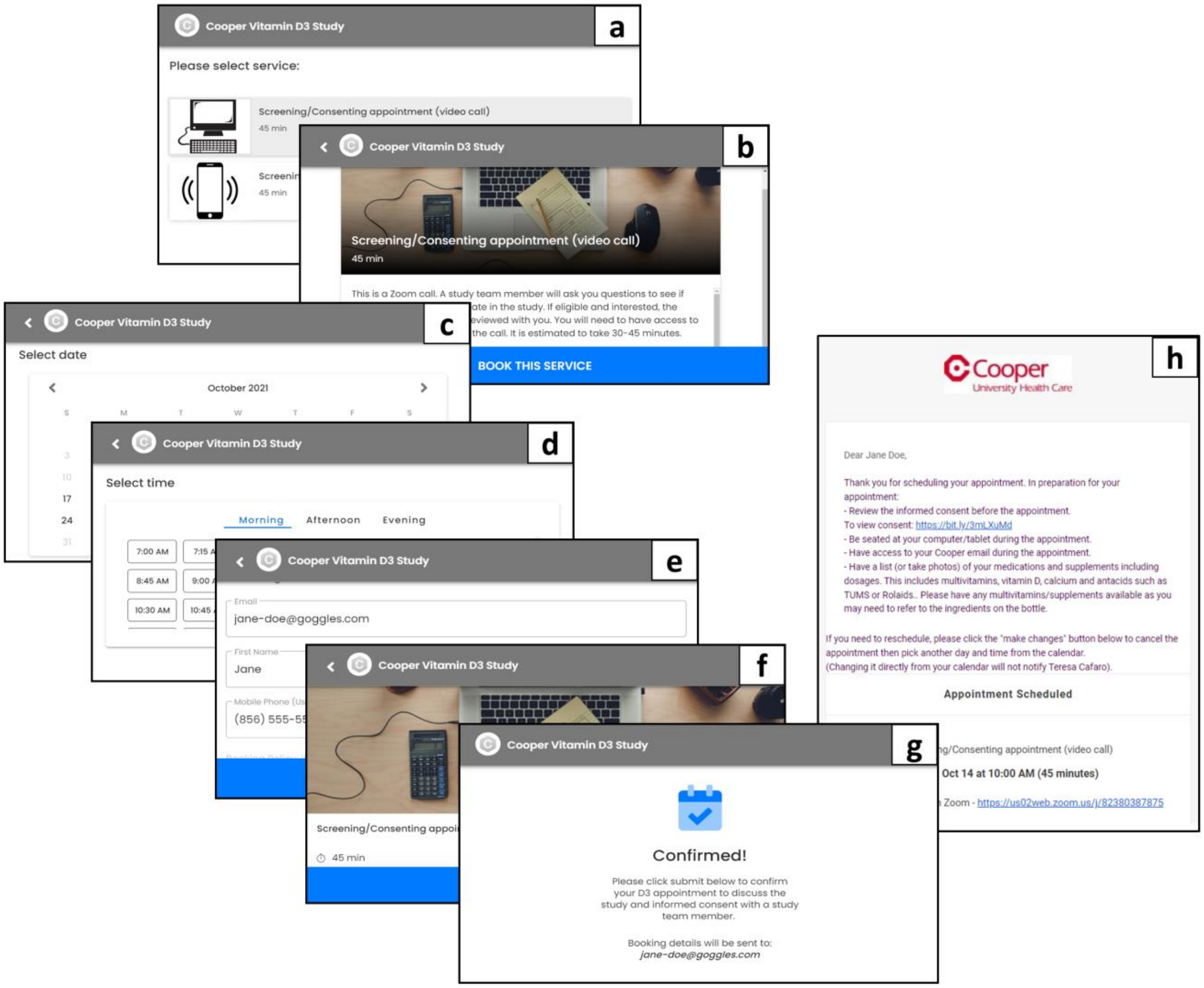

Supplementary Figure 2. Study information survey - Electronic appointment scheduling.

The Study Information Survey (Supplementary Figure 1) included an electronic appointment scheduler for subjects to schedule their screening and consent appointment at an available time slot. We illustrate here an overview of the scheduling process for the subject. A third party HIPAA-compliant appointment scheduler (vCita, Bellevue, WA, USA) which was developed for telemedicine appointments was used. The scheduling application was integrated within REDCap using API (Application Programming Interface). Both telephone and video call (Zoom Video Communications, San Jose, CA, USA) appointments were offered (Panels a, b). The scheduling application allowed for study team members to adjust their availability for appointment assignments (not shown). After a potential subject booked a screening appointment (Panels c, d, e, f, g), both the potential subject and study team member were sent an automated calendar invite for the appointment with the relevant contact information or video call link (Panel h). The study team member was also informed of scheduled appointment via text. In addition the potential subject was automatically sent a PDF copy of the informed consent form (without signature section) for their review before the screening appointment as well as instructions on how to prepare for the screening appointment. Instructions for the appointment included reviewing the informed consent form, having information on any medications or supplements they were taking available, and being seated at a

computer/tablet/smartphone with access to internet and email. The appointment scheduler was set to send reminder emails 24 hours and 30 minutes prior to each appointment.
