# Supplementary material for: Remote and semi-automated methods to conduct a decentralized randomized clinical trial
Source: J Clin Transl Sci. 2023 Jun 7;7(1):e153. doi: 10.1017/cts.2023.574 (PMC10388435; doi:10.1017/cts.2023.574)
Supplement: Supplementary file 1 [file S2059866123005745sup001.zip › suppl_data/S2059866123005745sup003.pdf]

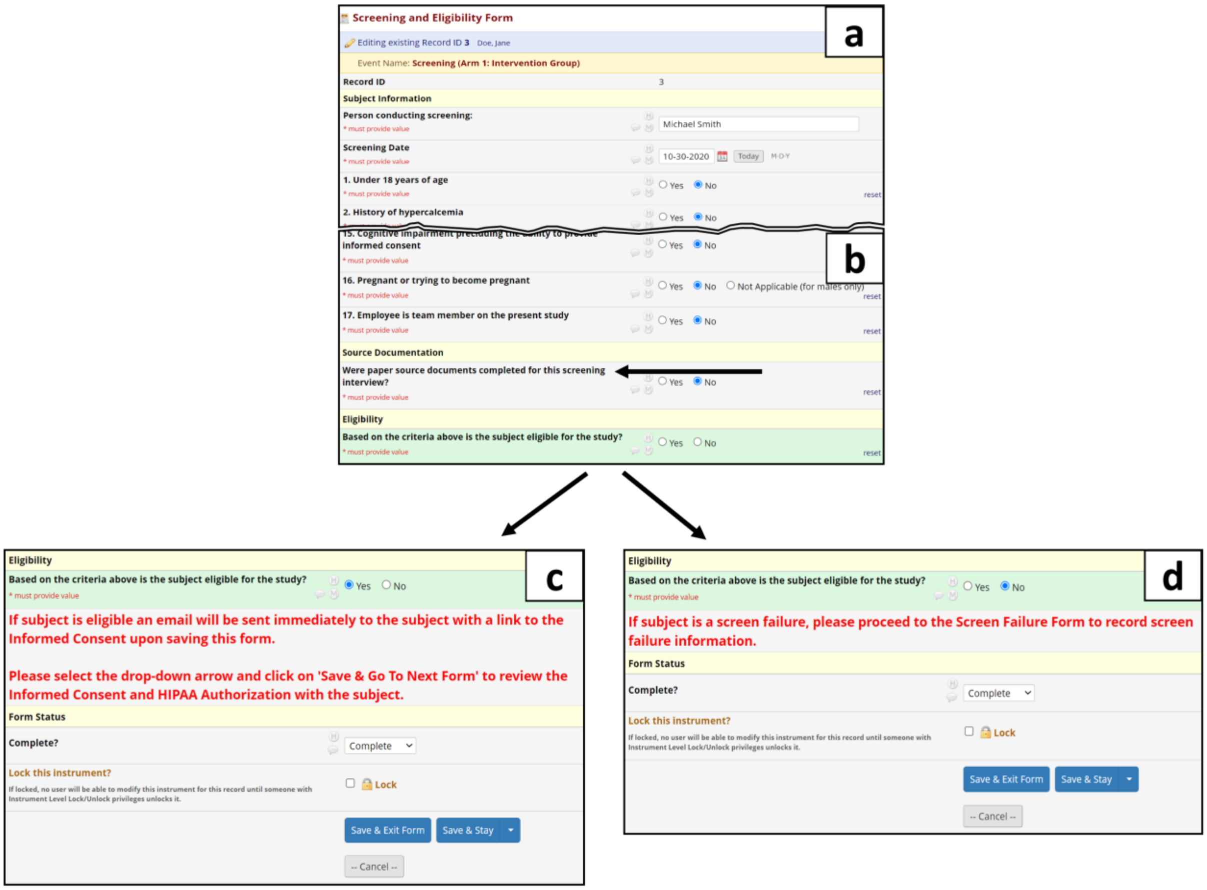

Supplementary Figure 3. Screening and eligibility procedures.

Panel a: Study team screened subjects with the aid of the Screening and Eligibility form. A Screening and Eligibility Phone Script was provided for use as source documentation or as a reference tool. Panel b: Team member chose direct data entry or paper source documentation. The eligibility question, however, must be answered “yes” at the time of screening to move onto the electronic consenting process. If source documents are used (=yes), instructions dynamically appear (not shown): “Please upload source documents into the REDCap File Repository. Your paper source documents will need to be sent to Cooper Anesthesiology Department for filing/storage at the end of the screening and enrollment period.” Panel c: Utilizing REDCap branching logic, specific instruction is provided to study team if subject is eligible (=yes) and wishes to review/sign the informed consent. Panel d: If subject is not eligible (=no) instruction to complete the screen failure form dynamically appears.
