# Supplementary material for: Remote and semi-automated methods to conduct a decentralized randomized clinical trial
Source: J Clin Transl Sci. 2023 Jun 7;7(1):e153. doi: 10.1017/cts.2023.574 (PMC10388435; doi:10.1017/cts.2023.574)
Supplement: Supplementary file 1 [file S2059866123005745sup001.zip › suppl_data/S2059866123005745sup005.pdf]

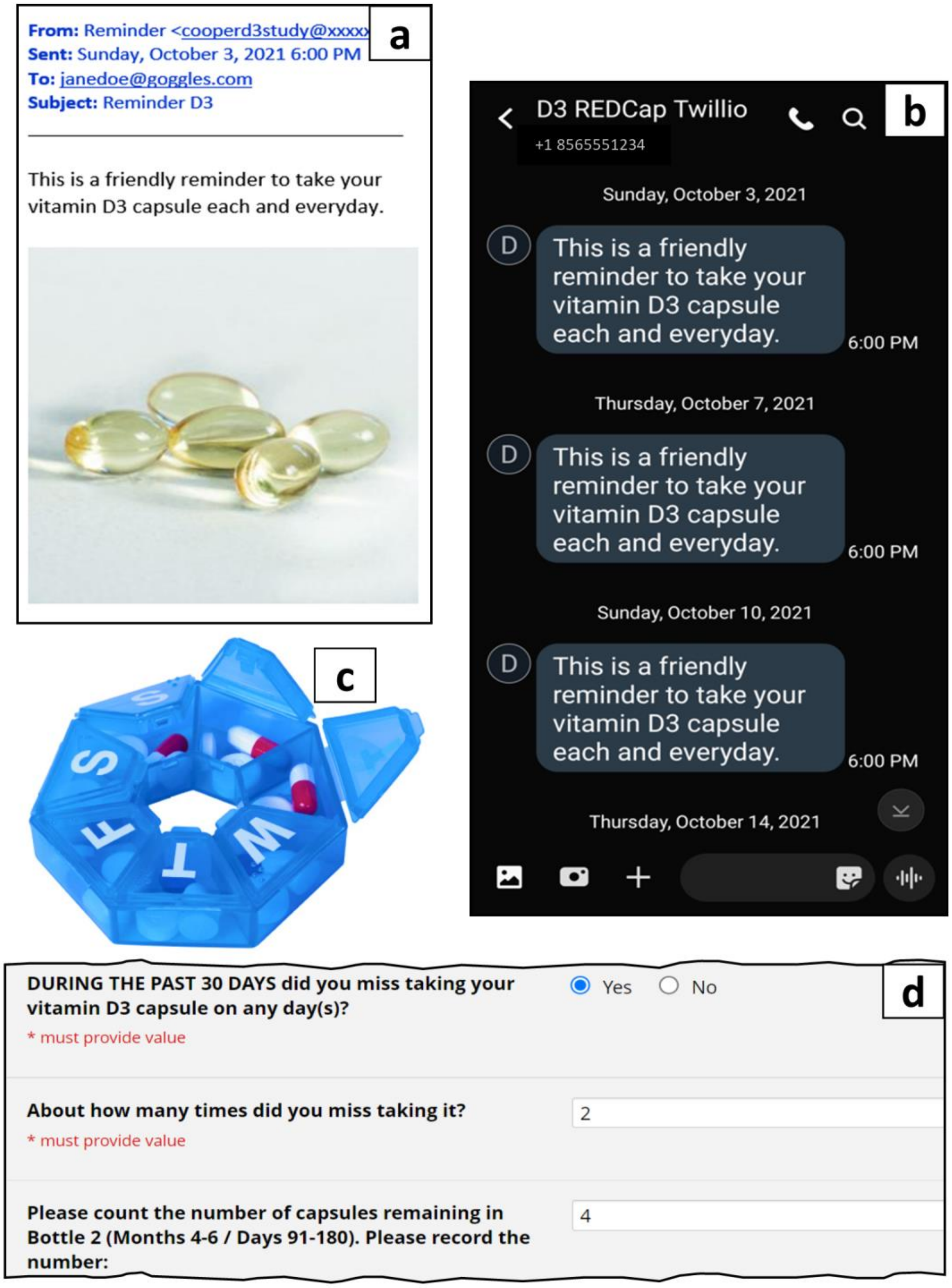

Supplementary Figure 4. Study product adherence.

To increase adherence automated email reminders (Panel a) and mobile text messages (Panel b) were sent Thursday and Sunday evenings during the subjects' participation in the study. With the first bottle of study product, subjects were shipped 7-day pill boxes (Pill Thing, Ellisville, MO, USA) to help them remember to take the capsules (Panel c). Finally, on the monthly surveys subjects were asked if they missed taking any capsules (Panel d). In addition, subjects counted and reported remaining pills in each of the 3 bottles at months 3, 6 and 9. REDCap reports (not shown) were used to contact any subject with less than 70% adherence rate to determine errors or other reasons for low adherence rates.
