# Supplementary material for: Remote and semi-automated methods to conduct a decentralized randomized clinical trial
Source: J Clin Transl Sci. 2023 Jun 7;7(1):e153. doi: 10.1017/cts.2023.574 (PMC10388435; doi:10.1017/cts.2023.574)
Supplement: Supplementary file 1 [file S2059866123005745sup001.zip › suppl_data/S2059866123005745sup007.pdf]

Comparison of Efforts in Remote and In-Person Trials

The following table is a comparison of our remote trial using electronic platforms versus the theoretical conduct of the same trial had it been conducted in person without the electronic means used in our study. Based on professional experience, the table displays which trial type (remote or in-person) was more efficient in time and manpower in the completion of various trial-related tasks.

Trial-Related Task*	Comparison of remote vs in-person trial for the least time and least manpower to conduct trial tasks	Remote	In-Person
Construction of Forms and Surveys for Data Collection	Time: Same<br>Manpower: Same	Creating the content of surveys takes the same amount of time as for In-Person.	Creating the content of surveys takes the same amount of time as for Remote.
Distribution of Surveys for Data Collection	Time: Remote<br>Manpower: Remote	Survey links are emailed. Data is instantaneously captured. Data entry into a database is automated. No paper is involved.	Surveys are printed, placed in envelopes, mailed; tracked; received and completed by subject; subject returns survey by mail; survey answers are data entered by hand.
Appointment Scheduling	Time: Remote<br>Manpower: Remote	Appointments are self-scheduled via automated appointment scheduling system. Rescheduling or cancellations are done via the electronic scheduling system.	Appointments are made by phone or email. Personnel is needed for scheduling, rescheduling or cancellations.
Screening	Time: Remote<br>Manpower: Same	Screening is performed via phone or video. Data capture and data entry into database can occur simultaneously (i.e., direct data entry is acceptable without the need for source documents).	Screening performed over phone. Paper source documents used. Data entry into database occurs by hand.
Consenting	Time: Remote<br>Manpower: Remote	Obtain consent via phone or video. Consent is electronically signed. Subject downloads auto-generated PDF of consent. Consent is automatically filed in electronic repository.	Obtaining informed consent is completed in person. Consent is signed on paper. Photocopy of consent is made for the subject. Paper consent is filed.
Dispensing Study Product	Time: Same<br>Manpower: Remote	Product labelled, packaged and mailed to subject. Packages taken to shipment vendor.	Product labelled, packaged and handed to subject. Personnel from Pharmacy and Receiving department of institution involved.

Trial-Related Task*	Comparison of remote vs in-person trial for the least time and least manpower to conduct trial tasks	Remote	In-Person
Source document Filing	Time: Remote<br>Manpower: Remote	Direct data entry used by most team members.	Paper source documents used to collect data which must be entered into the database and filed.
Safety Monitoring	Time: Remote<br>Manpower: Remote	Instead of manually reading each monthly survey on 299 subjects, surveys were manually read only if an alert was triggered. Thus instead of 4,142 surveys being read manually, 308 were read manually.	Manual review of surveys for potential adverse events would be required for every survey inviting error or missed adverse events. Manual review 4,142 surveys completed over the course of the study.
Adverse Event Monitoring and Follow-up	Time: Remote<br>Manpower: Remote	Preparation for AE follow up contact involves navigation to Subject Summary form displaying subject name, phone, medical history, past survey responses, reports and follow-up of all adverse events. 308 AEs were reported in 185 subjects.	Preparation for AE follow up contact involves manual file lookup to obtain patient contact information, medical history reported at baseline, and previous adverse events reported. 388 AEs were reported in 185 subjects.

* Development time for the remote and electronic aspects of the trial are not included as these activities are known to be time-consuming. In addition, the aim of our article is to share our XML file, methods and tools so one can reduce their learning curve and their development time. Our development time included, but is not limited to, forms and survey development in REDCAP; conditional logic for automated survey invitations and alerts, researching and interviewing potential vendors for electronic appointment scheduling; learning and integrating the electronic appointment scheduling system; testing and training study team on the appointment scheduler; and repeated testing of the system prior to deployment.
