# Supplementary material for: Remote and semi-automated methods to conduct a decentralized randomized clinical trial
Source: J Clin Transl Sci. 2023 Jun 7;7(1):e153. doi: 10.1017/cts.2023.574 (PMC10388435; doi:10.1017/cts.2023.574)
Supplement: Supplementary file 1 [file S2059866123005745sup006.pdf › suppl_data/S2059866123005745sup006.pdf]

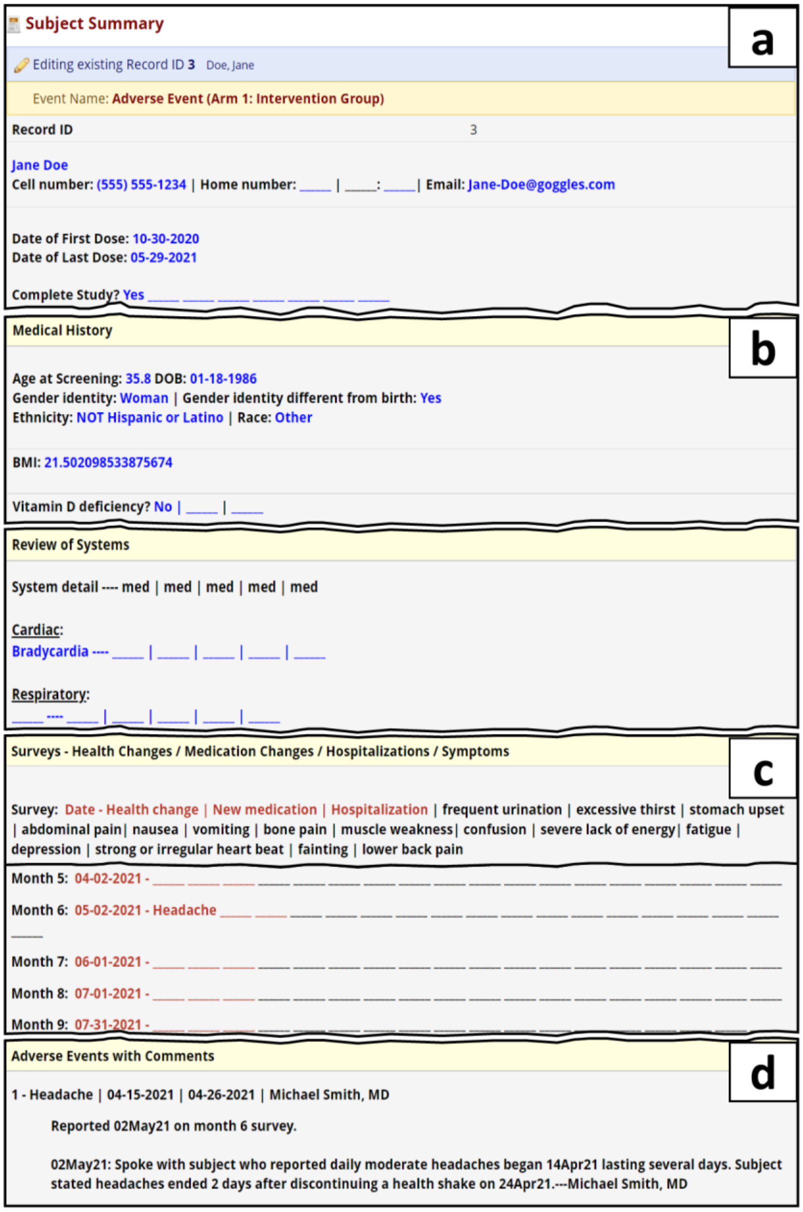

Supplementary Figure 5. Subject summary.

Utilizing the piping capability in REDCap, subject data was "piped" into this form from other surveys and forms (e.g., PHI, Demography and Medical Hx, monthly surveys, Adverse Events) to provide a summary of subject information. The summary was primarily used for reference when following up with subjects on adverse events. Panel a: The summary form streamlined communication logistics by including the subject's name, email and phone number, along with date of first dose, last dose and subject status. Panel b: Demographics, BMI and subject-reported medical history was shown to provide background medical information. Panel c: Concise descriptors (e.g., frequent urination) for each survey question acts as a header for the piped survey responses below the header (i.e., survey name, date of survey and subject entries, if any). Panel d: Each adverse event term is listed with start/end date and additional comments.
